# Supplementary material for: Functional impact of splicing variants in the elaboration of complex traits in cattle
Source: Nat Commun. 2025 Apr 24;16:3893. doi: 10.1038/s41467-025-58970-5 (PMC12022281; doi:10.1038/s41467-025-58970-5)
Supplement: Supplementary file 1 — Supplementary Information [file 41467_2025_58970_MOESM1_ESM.pdf]

Supplementary Information

Functional impact of splicing variants in the elaboration of complex traits in cattle

Mathieu Charles[1,2#], Nicolas Gaiani[1#], Marie-Pierre Sanchez[1#], Mekki Boussaha[1], Chris Hozé[1,3], Didier Boichard[1], Dominique Rocha[1], Arnaud Boulling[1,*]

[1] Université Paris-Saclay, INRAE, AgroParisTech, GABI, 78350 Jouy-en-Josas, France
[2] INRAE, SIGENAE, 78350 Jouy-en-Josas, France
[3] ELIANCE, 75012 Paris, France
* Corresponding author (mail: arnaud.boulling@inrae.fr)
# equal contribution

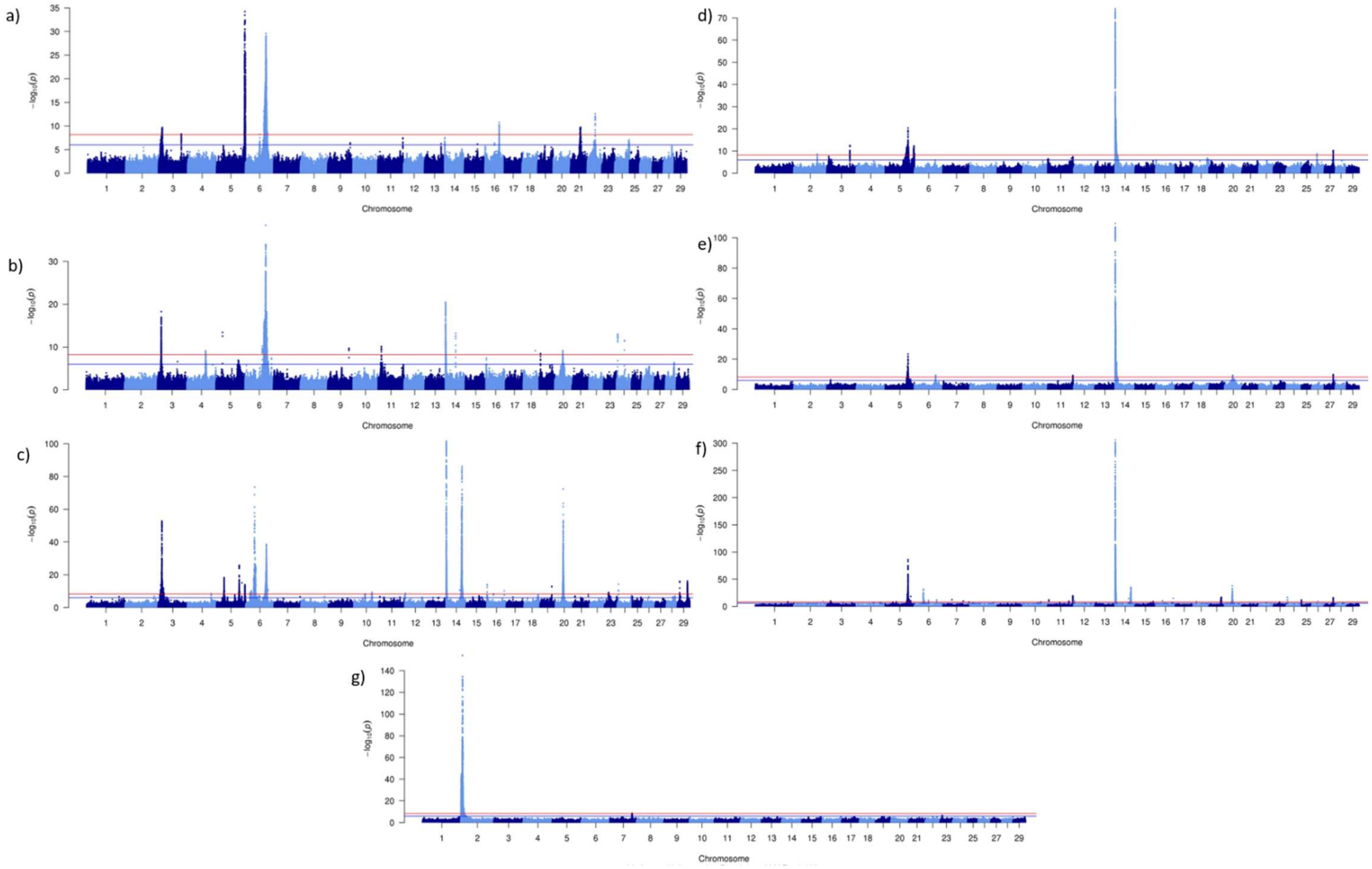

Supplementary Figure 1. Manhattan Plots of GWAS: -log10(P-value) of the effects of variants plotted against their position on *Bos taurus* autosomes. a) Milk protein content in Montbéliarde, **b)** Milk protein content in Normande, **c)** Milk protein content in Holstein, **d)** Milk fat content in Montbéliarde, **e)** Milk fat content in Normande, **f)** Milk fat content in Holstein, and **g)** muscularity score at month 30 in Charolaise. Source data are provided as a Source Data file.

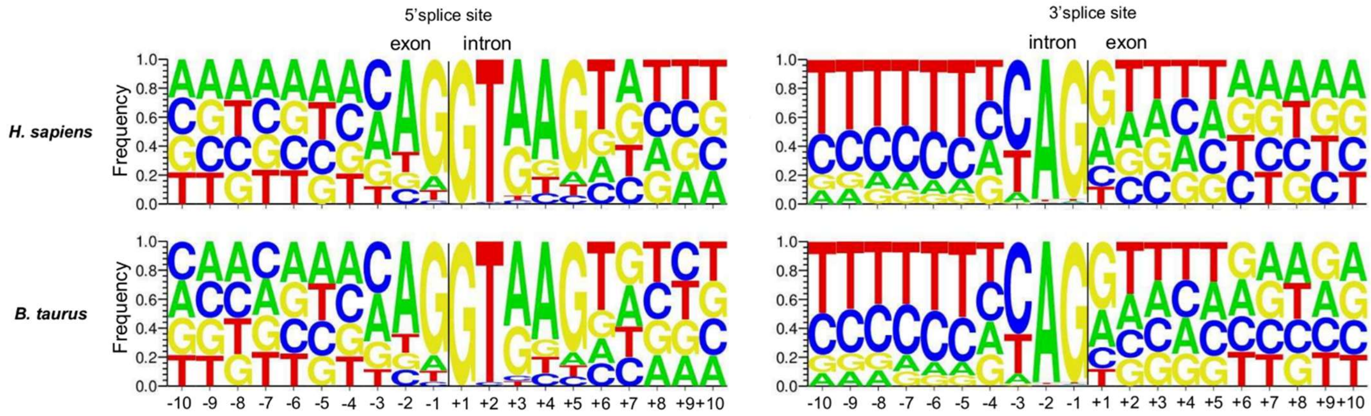

Supplementary Figure 2. Comparison of splice site sequences from human and cow. Pictograms display the frequency of each nucleotide at each position from -10 to +10 relative to the splice site in the 5' splice site (donor) and the 3' splice site (acceptor) regions in human and cow genes.

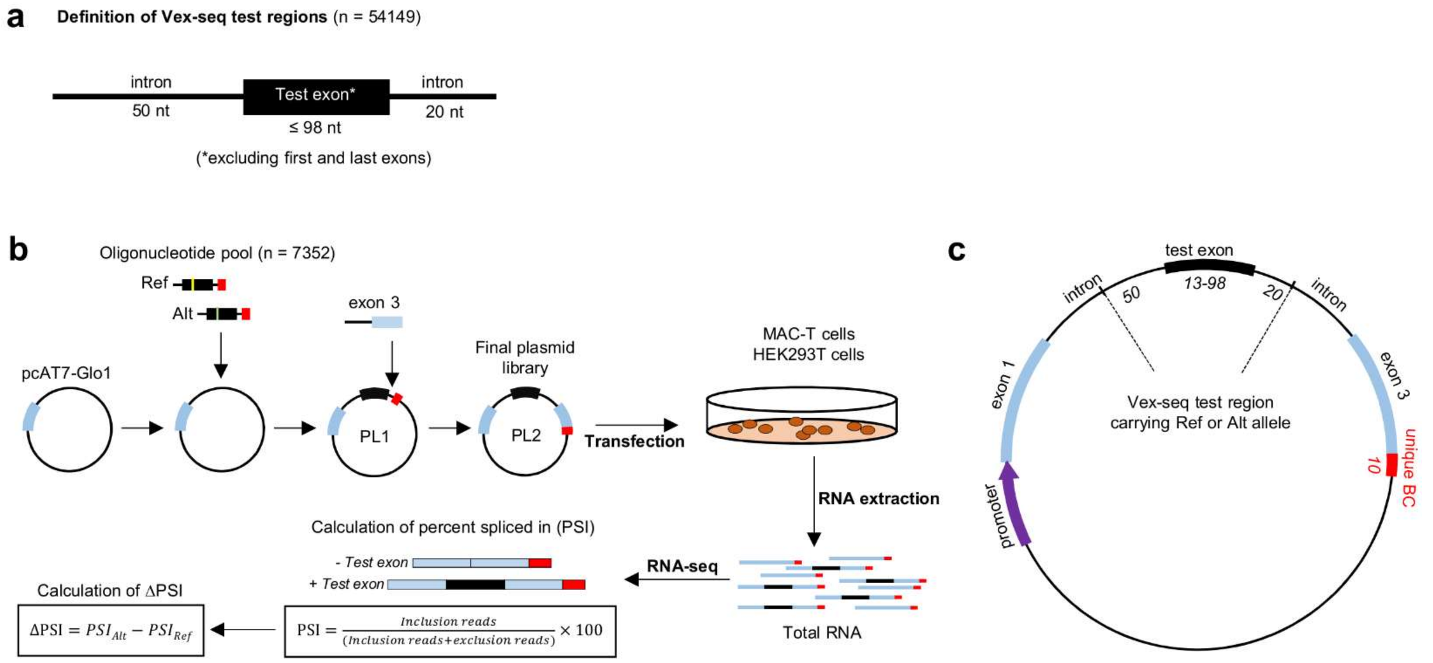

Supplementary Figure 3. Experimental workflow for the Vex-seq method. a) The regions that can be analysed using the Vex-seq method are limited in size. Therefore, the regions considered in our analysis (Vex-seq test regions) correspond only to exons of 98 nt or less in length and their flanking introns, 50 nt upstream and 20 nt downstream. The first and last exons of genes are excluded; in addition, the sequence tested must not contain any *Mfe*I or *Spe*I restriction site. **b)** The preparation of the final Vex-seq plasmid library involves a two-step subcloning of the test region into a reporter plasmid. First, an oligonucleotide pool of test regions is cloned into the pcAT7-Glo1 plasmid to obtain the Plasmid Library 1 (PL1). Note that each oligonucleotide carrying the test region sequence also contains a unique barcode (BC) of 10 nt length (coloured in red). A third exon is then cloned into the constructs between the test region and the BC to reconstitute the final Plasmid Library (PL2) made of complete functional minigenes. This arrangement strategically positions the BC, which identifies the specific sequence near the transcript's end. After transfection of these plasmids into cultured cells, a transcript is generated. This transcript may not necessarily contain the variant being tested, but it does carry the unique barcode associated with this latter. RT-PCR is used to generate cDNAs which are then sequenced by RNA-seq using the MiSeq platform. Based on BC identification, reads including or excluding test exons are counted in order to calculate PSI and ΔPSI for each variant. See supplementary references[1,2] for more details on the Vex-seq method. **c)** Map constructs constituting PL2.

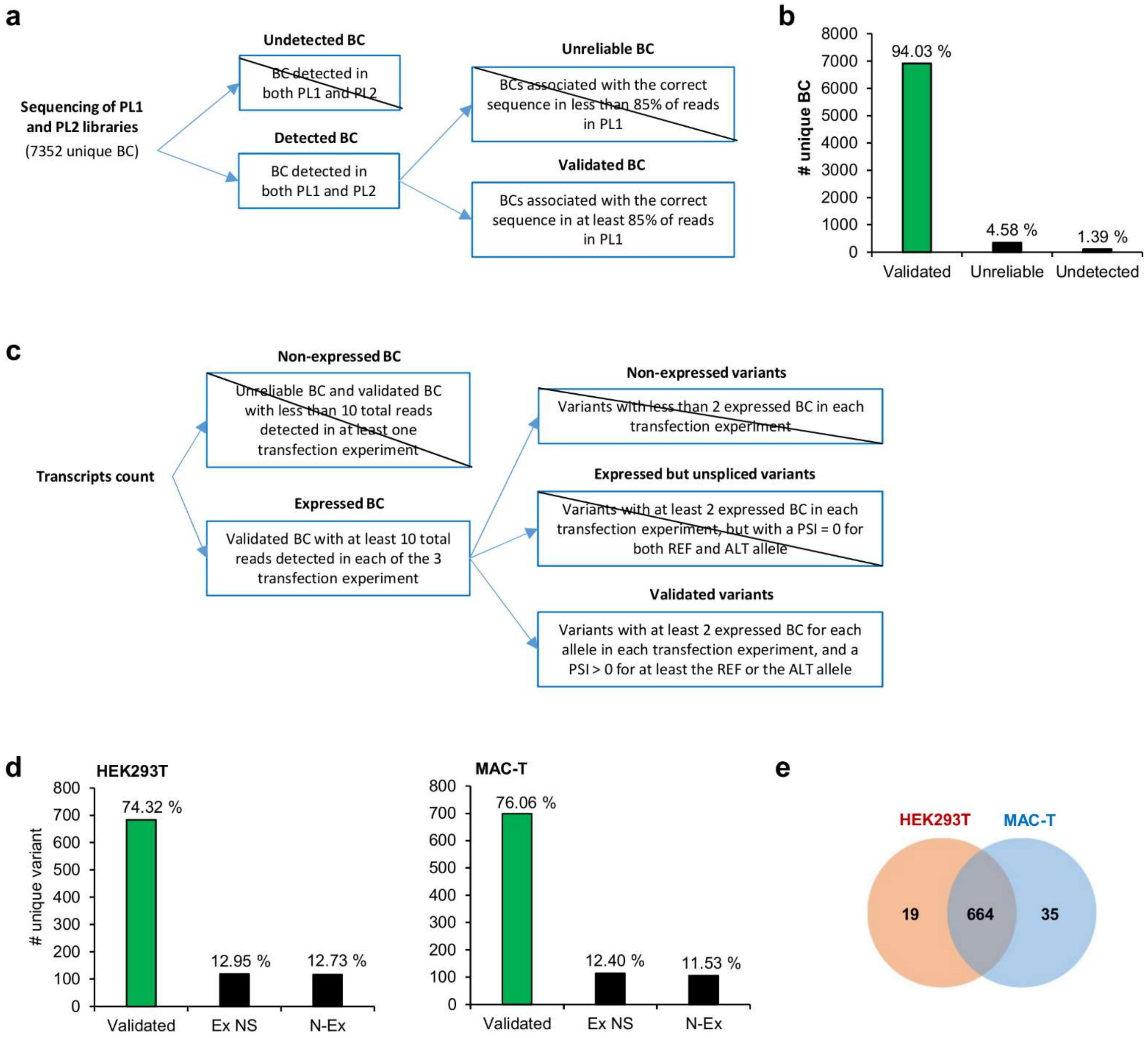

Supplementary Figure 4. Vex-seq quality control. a, b) Quality control of plasmid libraries by high-throughput sequencing. The absence of BC sequence alteration was checked in each of the 7352 constructs in PL1 and PL2. BC absent or containing synthesis errors were disregarded as well as BC associated with a low percentage (<85%) of correct reads in PL1. **c, d)** Filtering process to select interpretable variants in transcripts analysis. BC with less than 10 reads detected in RNA-seq were disregarded. Only variants with at least 2 expressed BC for each allele in each transfection experiment and a PSI > 0 for at least the REF or the ALT allele were considered for the calculation of ΔPSI. Ex NS, expressed but not spliced; N-Ex, non-expressed. **e)** Overlap between validated variants in HEK293T and MAC-T. Source data are provided as a Source Data file.

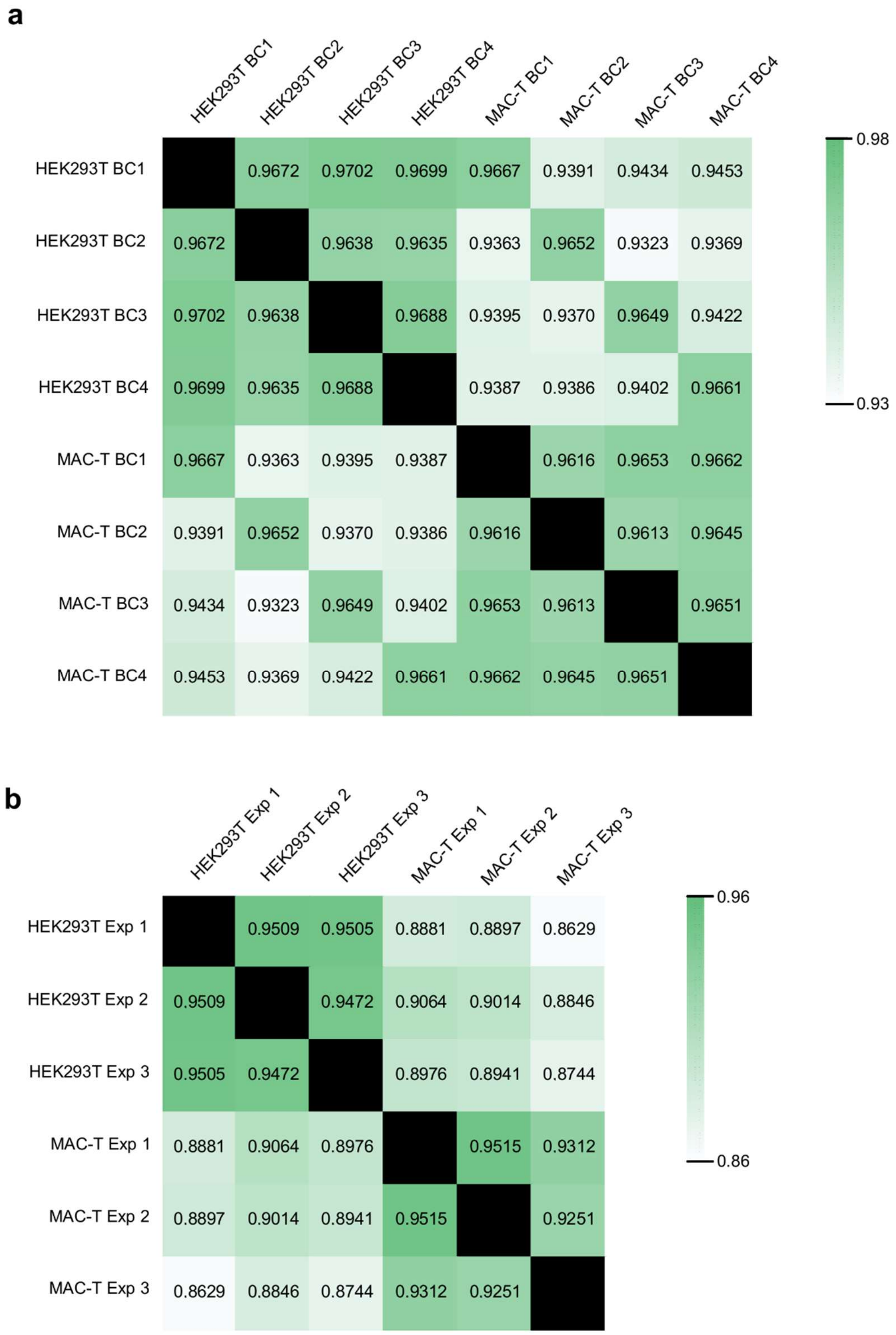

Supplementary Figure 5. Reproducibility of splicing outcomes. a) Correlation plot of average PSI value for each BC replicate in HEK293T and MAC-T cells and **b)** correlation plot of average PSI value for each biological replicate in HEK293T and MAC-T cells. The Pearson correlation coefficient is indicated, and dark green represents higher values.

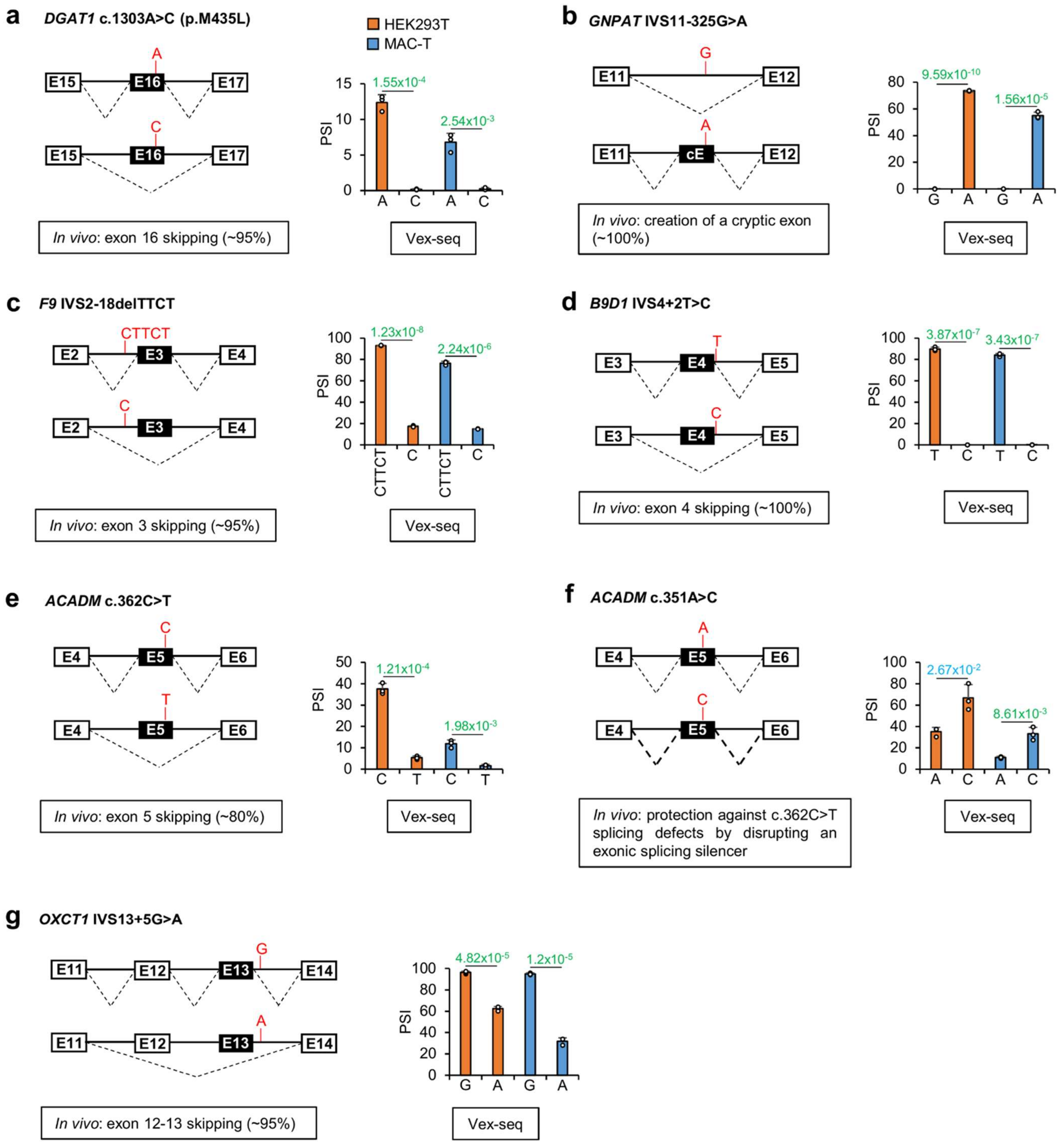

Supplementary Figure 6. Vex-seq analysis results are consistent with the functional impact of bovine and human splicing variants described *in vivo*. Splicing defects are depicted for **a, b)** bovine and **c-g)** human SDV characterized *in vivo and* reported in the literature. PSI measured in HEK293T and MAC-T cells are shown for each variant allele. Histograms represent mean values of three transfections with error bars of standard deviation. Each white point represents one transfection. The p-values (two-tailed student's t-test) have been calculated and the associated FDR are indicated. FDR<0.05 are in blue and FDR<0.01 are in green. Supplementary references: *DGAT1* c.1303A>C (p.M435L)[3], *GNPAT* IVS11-325G>A[4], *F9* IVS2-18delTTCT[5], *B9D1* IVS4+2T>C[6], *ACADM* c.362C>T[7], *ACADM* c.351A>C[7], *OXCT1* IVS13+5G>A[8]. Source data are provided as a Source Data file.

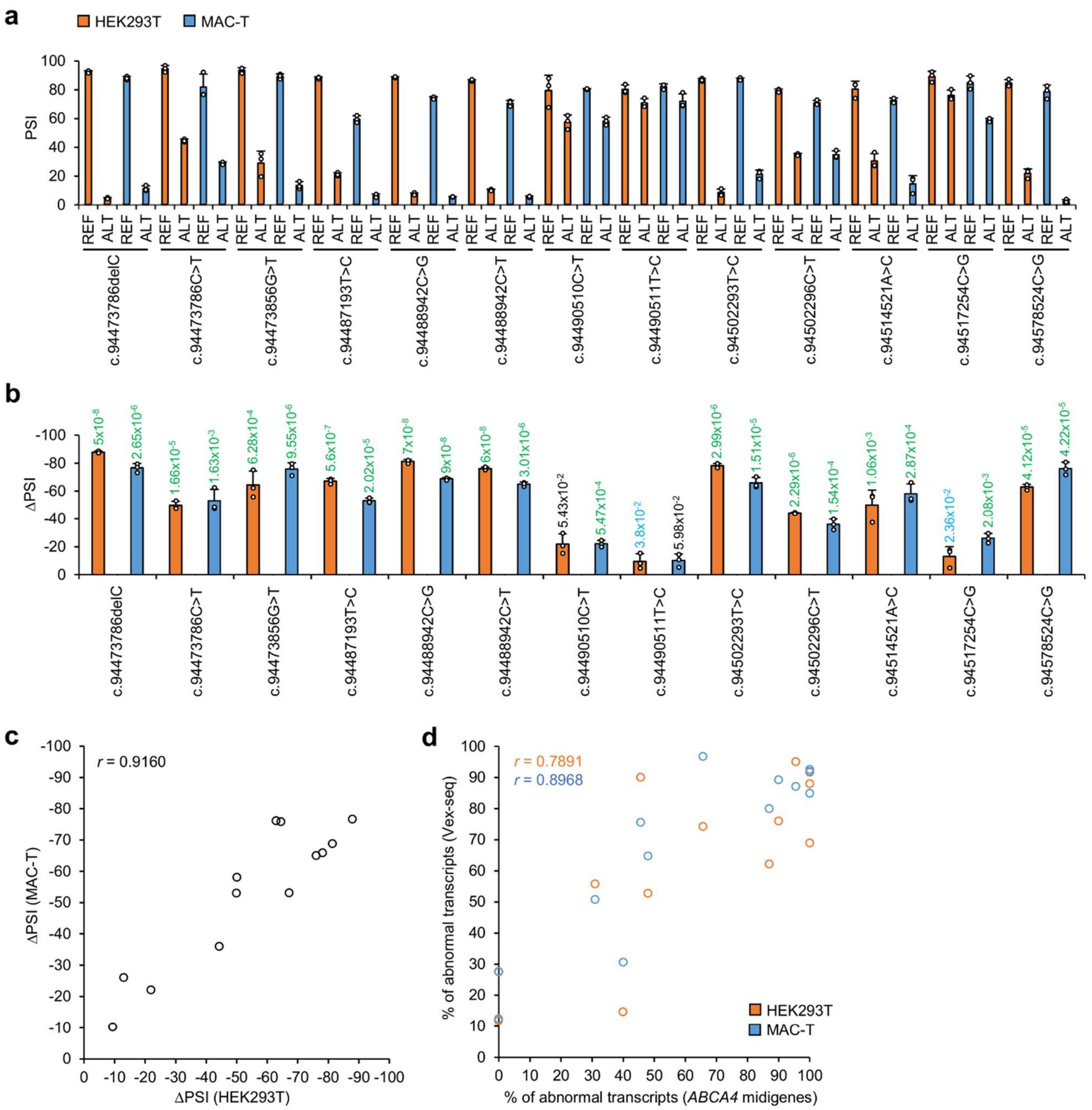

e

	FDR<0.05		FDR<0.01	
	HEK293T	MAC-T	HEK293T	MAC-T
TP	11	11	10	11
TN	1	1	2	1
FP	1	1	0	1
FN	0	0	1	0

f

	FDR<0.05			FDR<0.01		
	HEK293T	MAC-T	Average	HEK293T	MAC-T	Average
Sensitivity (%)	100	100	**100**	90.9	100	**95.45**
Specificity (%)	50	50	**50**	100	50	**75**

Supplementary Figure 7. Assessing the Vex-seq performance using a dataset of 13 human *ABCA4* variants validated by midigene[9]. a) PSI of *ABCA4* variant alleles measured by Vex-seq. Histograms represent mean values of three transfections with error bars of standard deviation. Each white point represents one transfection. **b)** ΔPSI calculated for variants presented in (a). The p-values (two-tailed student's t-test) have been calculated and the associated FDR are indicated. FDR<0.05 are in blue and FDR<0.01 are in green. **c)** Scatter plot of ΔPSI in HEK293T versus MAC-T cells. *r*, Pearson correlation coefficient. **d)** Scatter plot of the percentage of abnormal transcript measured using midigenes versus Vex-seq. **e)** Number of true positive (TP), false positive (FP), true negative (TN), and false negative (FN) in HEK293T and MAC-T cells depending on the FDR threshold and using *ABCA4* variants analyzed by midigene as a benchmark. **f)** Calculation of the sensitivity and specificity of Vex-seq according to (e). Source data are provided as a Source Data file.

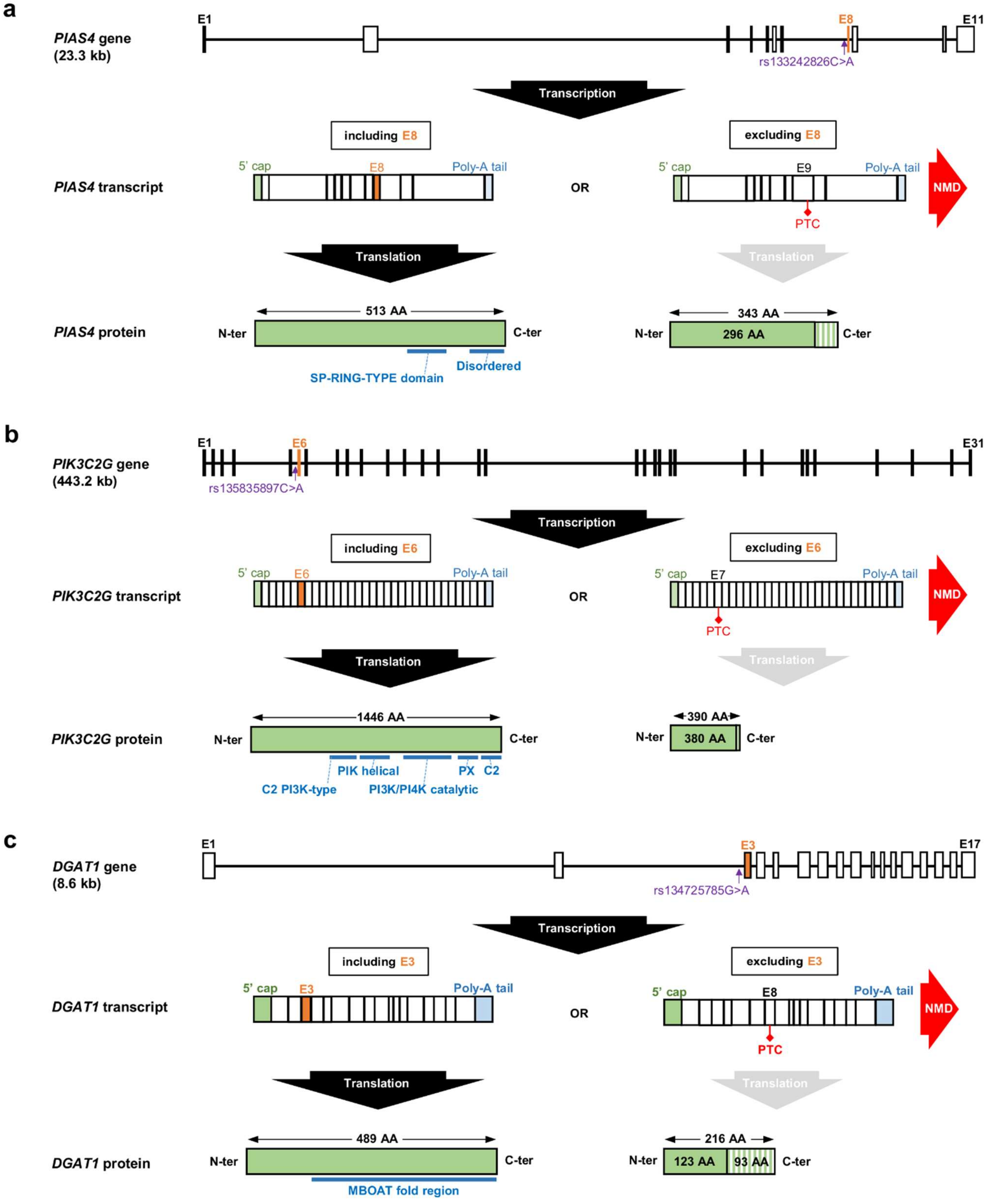

Supplementary Figure 8. Predicted consequences of rs133242826, rs135835897 and rs134725785 on *PIAS4*, *PIK3C2G* and *DGAT1* function, respectively. The prediction was made on the structure of the transcript and protein for each gene, considering transcript isoforms including or not the test exon whose inclusion is modulated by the variant. Where appropriate, the presence of a premature termination codon (PTC) was indicated, as well as degradation by the non-sense mRNA decay (NMD). The main functional regions and domains defined by UniProt that were lost after protein truncation are underlined in blue. Abnormal amino acids (AA) due to reading frame shift are crosshatched. Ensembl transcript ID and UniProt ID used for prediction: *PIAS4*, ENSBTAT00000001000, A4FV15; *PIK3C2G*, ENSBTAT00000043102, E1B8D1; *DGAT1*, ENSBTAT00000037423, Q8MK44.

Trait[1]	Charolaise	Holstein	Montbéliarde	Normande	Total
MY	-	8,100 (23)	354 (6)	1,006 (11)	9,460 (40)
FY	-	9,250 (19)	2,205 (7)	596 (8)	12,051 (34)
PY	-	4,992 (20)	315 (14)	457 (9)	5,764 (43)
FC	-	20,518 (31)	10,074 (30)	11,518 (18)	42,110 (79)
PC	-	34,140 (66)	25,334 (27)	19,751 (23)	79,225 (116)
ICFI	-	4,700 (19)	1,913 (6)	13 (5)	6,626 (30)
HCR	-	568 (7)	14 (4)	698 (7)	1,280 (18)
CCR	-	5,134 (14)	0 (0)	13 (3)	5,147 (17)
SCS	-	2,343 (22)	282 (13)	78 (4)	2,703 (39)
MAST	-	3,185 (11)	1063 (3)	1 (1)	4,249 (15)
W18	3,395 (11)	-	-	-	3,395 (11)
W24	4,156 (11)	-	-	-	4,156 (11)
MS30	17,044 (11)	-	-	-	17,044 (11)
SS30	8,652 (23)	-	-	-	8,652 (23)
TB30	10,406 (21)	-	-	-	10,406 (21)
THIGHS	-	-	141 (11)	1,846 (8)	1,987 (19)
WITHER	-	-	425 (10)	501 (7)	926 (17)
CW	32 (5)		0	0 (0)	32 (5)
AS	15 (4)		27 (2)	15 (2)	57 (8)
CG	3,249 (9)		185 (5)	19 (2)	3,453 (16)
Total	46,949 (95)	92,930 (232)	42,332 (138)	36,512 (108)	218,723

Supplementary Table 1. Number of candidate variants (-log(p-value)>6) and number of QTL (in parentheses) in each trait x breed GWAS combination. [1]See Table 1.

Breed	Number of sequenced animals
Holstein	1059
Brown Swiss	231
Fleckvieh	158
Charolais	147
Simmental	125
Limousin	104
Montbeliarde	63
Modern Danish Red	55
Normande	45
Holstein-Friesian	42
Blonde d'Aquitaine	41
Swedish Red	35
Salers	25
Maine-Anjou	22
Holstein Red	20
Traditional Danish Red	15
Tarentaise	12
Belgian Blue	10
Abondance	9
Aubrac	9
Rouge Des Pres	9
Meuse Rhine Yssel	5
Vosgienne	4
Danish Red Dairy	3
Nordic Red Cattle	2
Parthenaise	2
Red Dairy	2
Crossbreed (Holstein 62.5%; Montbéliarde 25%; Jersey 12.5%)	1
Total	2255

Supplementary Table 2. Composition of the multibreed reference population used for imputation of genotypes at the sequence level

Name	Sequence (5'-3')
Oligo-F	CTGACTCTCTCTGCCTC
Oligo-R	TAAACGGGCCCTCTAGA
Exon3-MfeI-F	GTGTGGAAGTCTCAGGATCG
Exon3-XbaI-R	AACGGGCCCTCTAGAGC
PL1-F	acactctttccctacacgacgctcttccgatctCCACTGACTCTCTCTGCCTC
PL2-F	acactctttccctacacgacgctcttccgatctAGCAGCTACAATCCAGCTACCA
Plasmid-R	gtgactggagttcagacgtgtgctcttccgatctAGCGGGTTTAAACGGGCCCT
i5-UDI0001-F	AATGATACGGCGACCACCGAGATCTACACAGCGCTAGACACTCTTTCCCTACACGACGCTCTTCCGATCT
i7-UDI0001-R	CAAGCAGAAGACGGCATACGAGATCCGCGGTTGTGACTGGAGTTCAGACGTGTGCTCTTCCGATCT
i5-UDI0002-F	AATGATACGGCGACCACCGAGATCTACACGATATCGAACACTCTTTCCCTACACGACGCTCTTCCGATCT
i7-UDI0002-R	CAAGCAGAAGACGGCATACGAGATTTATAACCGTGACTGGAGTTCAGACGTGTGCTCTTCCGATCT
VS-F1	AATGATACGGCGACCACCGAGATCTACACCGACTTTGACACTCTTTCCCTACACGACGCTCTTCCGATCTGGCAAGGTGAACGTGGATGAAG
VS-F2	AATGATACGGCGACCACCGAGATCTACACAGGCTGTCACACTCTTTCCCTACACGACGCTCTTCCGATCTNGGCAAGGTGAACGTGGATGAAG
VS-F3	AATGATACGGCGACCACCGAGATCTACACATGCCGAGACACTCTTTCCCTACACGACGCTCTTCCGATCTNNGGCAAGGTGAACGTGGATGAAG
i7-UDI0003-R	CAAGCAGAAGACGGCATACGAGATGGACTTGGGTGACTGGAGTTCAGACGTGTGCTCTTCCGATCT
i7-UDI0004-R	CAAGCAGAAGACGGCATACGAGATAAGTCCAAGTGACTGGAGTTCAGACGTGTGCTCTTCCGATCT
i7-UDI0005-R	CAAGCAGAAGACGGCATACGAGATATCCACTGGTGACTGGAGTTCAGACGTGTGCTCTTCCGATCT
i7-UDI0006-R	CAAGCAGAAGACGGCATACGAGATGCTTGTCAGTGACTGGAGTTCAGACGTGTGCTCTTCCGATCT

Supplementary Table 3. Primers name and sequence. Index sequence is underlined.

Supplementary references

1. Adamson, S. I., Zhan, L. & Graveley, B. R. Vex-seq: high-throughput identification of the impact of genetic variation on pre-mRNA splicing efficiency. *Genome Biol* **19**, 71 (2018).
2. Adamson, S., Zhan, L. & Graveley, B. *Functional Characterization of Splicing Regulatory Elements*. http://biorxiv.org/lookup/doi/10.1101/2021.05.14.444228 (2021) doi:10.1101/2021.05.14.444228.
3. Lehnert, K. *et al.* Phenotypic population screen identifies a new mutation in bovine DGAT1 responsible for unsaturated milk fat. *Sci Rep* **5**, 8484 (2015).
4. Boulling, A. *et al.* A bovine model of rhizomelic chondrodysplasia punctata caused by a deep intronic splicing mutation in the *GNPAT* gene. Preprint at https://doi.org/10.1101/2024.06.13.598642 (2024).
5. Van de Water, N. S., Tan, T., May, S., Browett, P. J. & Harper, P. Factor IX polypyrimidine tract mutation analysis using mRNA from peripheral blood leukocytes. *J Thromb Haemost* **2**, 2073–2075 (2004).
6. Hopp, K. *et al.* B9D1 is revealed as a novel Meckel syndrome (MKS) gene by targeted exon-enriched next-generation sequencing and deletion analysis. *Hum Mol Genet* **20**, 2524–2534 (2011).
7. Nielsen, K. B. *et al.* Seemingly neutral polymorphic variants may confer immunity to splicing-inactivating mutations: a synonymous SNP in exon 5 of MCAD protects from deleterious mutations in a flanking exonic splicing enhancer. *Am J Hum Genet* **80**, 416–432 (2007).
8. Hori, T. *et al.* Molecular basis of two-exon skipping (exons 12 and 13) by c.1248+5g>a in OXCT1 gene: study on intermediates of OXCT1 transcripts in fibroblasts. *Hum Mutat* **34**, 473–480 (2013).
9. Sangermano, R. *et al.* ABCA4 midigenes reveal the full splice spectrum of all reported noncanonical splice site variants in Stargardt disease. *Genome Res* **28**, 100–110 (2018).
