# Supplementary material for: Functional impact of splicing variants in the elaboration of complex traits in cattle
Source: Nat Commun. 2025 Apr 24;16:3893. doi: 10.1038/s41467-025-58970-5 (PMC12022281; doi:10.1038/s41467-025-58970-5)
Supplement: Supplementary file 2 — Description of Additional Supplementary Files [file 41467_2025_58970_MOESM2_ESM.pdf]

Description of Additional Supplementary Files

File Name: Supplementary Data 1

Description: Twenty four SDV described *in vivo* and associated with phenotypes in cattle

File Name: Supplementary Data 2

Description: Detailed functional information related to validated SDV used as positive controls

File Name: Supplementary Data 3

Description: Detailed functional information related to variants from Var.GWAS dataset

File Name: Supplementary Data 4

Description: Detailed functional information related to variants from Var.P dataset

File Name: Supplementary Data 5

Description: SDV identified using Vex-seq and associated phenotypes

File Name: Supplementary Data 6

Description: Colocalization between 38 SDV from Var.GWAS dataset and *cis*-e/sQTL SNP

File Name: Supplementary Data 7

Description: List of Vex-seq oligonucleotides related to variants analyzed in this study

File Name: Supplementary Data 8

Description: Ensembl Transcript ID and UniProt ID used to predict structural and functional consequences presented in Figure 8
