# Supplementary material for: Functional impact of splicing variants in the elaboration of complex traits in cattle
Source: Nat Commun. 2025 Apr 24;16:3893. doi: 10.1038/s41467-025-58970-5 (PMC12022281; doi:10.1038/s41467-025-58970-5)
Supplement: Supplementary file 11 — Reporting Summary [file 41467_2025_58970_MOESM11_ESM.pdf]

nature portfolio

Corresponding author(s): Arnaud Boulling (arnaud.boulling@inrae.fr)

Last updated by author(s): 27/03/25

Reporting Summary

Nature Portfolio wishes to improve the reproducibility of the work that we publish. This form provides structure for consistency and transparency in reporting. For further information on Nature Portfolio policies, see our Editorial Policies and the Editorial Policy Checklist.

Statistics

For all statistical analyses, confirm that the following items are present in the figure legend, table legend, main text, or Methods section.

n/a	Confirmed	
☐	☒	The exact sample size (*n*) for each experimental group/condition, given as a discrete number and unit of measurement
☐	☒	A statement on whether measurements were taken from distinct samples or whether the same sample was measured repeatedly
☐	☒	The statistical test(s) used AND whether they are one- or two-sided<br>*Only common tests should be described solely by name; describe more complex techniques in the Methods section.*
☐	☒	A description of all covariates tested
☐	☒	A description of any assumptions or corrections, such as tests of normality and adjustment for multiple comparisons
☐	☒	A full description of the statistical parameters including central tendency (e.g. means) or other basic estimates (e.g. regression coefficient) AND variation (e.g. standard deviation) or associated estimates of uncertainty (e.g. confidence intervals)
☐	☒	For null hypothesis testing, the test statistic (e.g. *F*, *t*, *r*) with confidence intervals, effect sizes, degrees of freedom and *P* value noted<br>*Give P values as exact values whenever suitable.*
☒	☐	For Bayesian analysis, information on the choice of priors and Markov chain Monte Carlo settings
☒	☐	For hierarchical and complex designs, identification of the appropriate level for tests and full reporting of outcomes
☐	☒	Estimates of effect sizes (e.g. Cohen's *d*, Pearson's *r*), indicating how they were calculated

Our web collection on statistics for biologists contains articles on many of the points above.

Software and code

Policy information about availability of computer code

Data collection	FImpute (v3) ; Minimac (v4) ; SAMtools (v0.0.18)
Data analysis	GCTA (v1.94.1) ; SpliceAI (v1.3.1) ; Pangolin (v1.0.2).<br>All custom scripts generated in this study have been deposited in the Recherche Data Gouv database [https://doi.org/10.57745/UO9T9O].

For manuscripts utilizing custom algorithms or software that are central to the research but not yet described in published literature, software must be made available to editors and reviewers. We strongly encourage code deposition in a community repository (e.g. GitHub). See the Nature Portfolio guidelines for submitting code & software for further information.

Data

Policy information about availability of data

All manuscripts must include a data availability statement. This statement should provide the following information, where applicable:

- Accession codes, unique identifiers, or web links for publicly available datasets
- A description of any restrictions on data availability
- For clinical datasets or third party data, please ensure that the statement adheres to our policy

The authors confirm that the summary of GWAS results, the results of SpliceAI and Pangolin predictions, in addition to the analysed data of the Vex-seq analysis, are available within the article. Source data are provided with this article.
The GWAS data generated in this study have been deposited in the Recherche Data Gouv database [https://doi.org/10.57745/UO9T9O]. The corresponding raw

phenotypic and genotypic data were produced for the purpose of bovine selection and belong to French farmers' organisations, which have given INRAE permission to use them for research purposes, excluding any transfer to third parties or public databases. They cannot therefore be made available to the public. Readers can request a research licence from Valogene (France, Paris) for the genotyping data and from France Génétique Elevage (France, Paris) for the phenotypic data. All MiSeq fastq files relative to the Vex-seq analysis and generated in this study have been deposited in the European Nucleotide Archive database under the accession code PRJEB87659 [https://www.ebi.ac.uk/ena/browser/view/PRJEB87659]. All files generated to run SpliceAI and Pangolin on bovine variants have been deposited in the Recherche Data Gouv database [https://doi.org/10.57745/UO9T9O].
eQTL and sQTL data from Liu et al.7 are available at the cattle Genotype-Tissue Expression atlas (https://cgtex.roslin.ed.ac.uk/). eQTL and sQTL data from Xiang et al.25 are available at (https://figshare.unimelb.edu.au/articles/dataset/eQTL_and_sQTL_from_16_cattle_tissues_linear_mixed_model_/19793047?file=35165539).

Research involving human participants, their data, or biological material

Policy information about studies with human participants or human data. See also policy information about sex, gender (identity/presentation), and sexual orientation and race, ethnicity and racism.

Reporting on sex and gender	N.A.
Reporting on race, ethnicity, or other socially relevant groupings	N.A.
Population characteristics	N.A.
Recruitment	N.A.
Ethics oversight	N.A.

Note that full information on the approval of the study protocol must also be provided in the manuscript.

Field-specific reporting

Please select the one below that is the best fit for your research. If you are not sure, read the appropriate sections before making your selection.

☒ Life sciences ☐ Behavioural & social sciences ☐ Ecological, evolutionary & environmental sciences

For a reference copy of the document with all sections, see nature.com/documents/nr-reporting-summary-flat.pdf

Life sciences study design

All studies must disclose on these points even when the disclosure is negative.

Sample size	No sample size calculation was performed neither for GWAS nor for Vex-seq analysis. GWAS were performed using genotypes and phenotypes (means of daughter performance for bulls) measured on 2,255 to 10,066 individuals, depending on the breed and on the trait. As shown in the manuscript, such sample sizes are large enough to detect QTL with moderate and strong effects. The number of variants functionally analysed was limited by Vex-seq capacity. A total of 919 variants was analysed, corresponding to 919 reference and 919 alternative test sequences each associated with 4 different barcodes for a total of 7352 test sequences. The False Discovery Rate was calculated simultaneously on all the variants. However, for the sake of clarity and to avoid overcomplicating the scientific issues addressed in this article, only a fraction of the variants (n=391) have been discussed. Results for the remaining variants will be presented and interpreted in future publications.
Data exclusions	For GWAS and Vex-seq analysis, variants were filtered out in accordance with the quality control described in the method section.
Replication	No attempt was made to assess the reproducibility of the experimental results.
Randomization	All input data for GWAS (phenotypes and genotypes) were commercial data generated for selection purpose and therefore randomization was not possible. Randomization was not applicable for Vex-seq analysis as only one plasmid library was used, in which all the tested sequence related to control and candidate variants were included.
Blinding	All input data for GWAS (phenotypes and genotypes) were data from commercial farms generated for selection purpose and therefore blinding was not possible. Blinding was not applicable for Vex-seq analysis as only one plasmid library was used, in which all the tested sequence related to control and candidate variants were included.

Reporting for specific materials, systems and methods

We require information from authors about some types of materials, experimental systems and methods used in many studies. Here, indicate whether each material, system or method listed is relevant to your study. If you are not sure if a list item applies to your research, read the appropriate section before selecting a response.

Materials & experimental systems

n/a	Involved in the study
☒	☐ Antibodies
☐	☒ Eukaryotic cell lines
☒	☐ Palaeontology and archaeology
☐	☒ Animals and other organisms
☒	☐ Clinical data
☒	☐ Dual use research of concern
☒	☐ Plants

Methods

n/a	Involved in the study
☒	☐ ChIP-seq
☒	☐ Flow cytometry
☒	☐ MRI-based neuroimaging

Eukaryotic cell lines

Policy information about cell lines and Sex and Gender in Research

Cell line source(s)	The HEK293T cells were provided by Sophie Dhorne-Pollet at INRAE ; GABI unit (FRANCE, Jouy-en-Josas). The MAC-T cells were provided by Kathrin Kober-Rychli at the University of Veterinary Medicine ; Institute of Food Safety, Food Technology and Veterinary Public Health (AUSTRIA, Vienna).
Authentication	None of the cell lines used were authenticated.
Mycoplasma contamination	The cell lines were not tested for mycoplasma contamination.
Commonly misidentified lines (See ICLAC register)	No commonly misidentified cell lines were used in the study.

Animals and other research organisms

Policy information about studies involving animals; ARRIVE guidelines recommended for reporting animal research, and Sex and Gender in Research

Laboratory animals	The study did not involved laboratory animals.
Wild animals	The study did not involved wild animals.
Reporting on sex	Each phenotype was measured in only one sex (male or female); therefore, each trait was analyzed within sex.
Field-collected samples	All input data for GWAS (phenotypes and genotypes) were data from commercial farms generated for selection purpose and therefore already existed before the study.
Ethics oversight	All analyses were performed using data from routine recording and genotyping of French cattle in commercial herds. We did not perform any experiments on animals and no ethical approval was required.

Note that full information on the approval of the study protocol must also be provided in the manuscript.

Plants

Seed stocks	N.A.
Novel plant genotypes	N.A.
Authentication	N.A.
